# Frailty in a Frailty Prevention Program Participants During COVID-19 pandemic: A Cross-Sectional Japanese Study

**DOI:** 10.1093/geroni/igab046.2999

**Published:** 2021-12-17

**Authors:** Takuya Kanamori, Mizue Suzuki, Tomoyoshi Naito, Keigo Inagaki, Hiroyuki Umegaki

**Affiliations:** 1 Hamamatsu University School of Medicine, Hamamatsu city, Shizuoka, Japan; 2 Nagoya University Graduate School of Medicine, Nagoya, Aichi, Japan

## Abstract

Objective: Health conditions of older adults have deteriorated during the COVID-19 pandemic. Few studies have reported on the frailty of this group of people. The study aimed to investigate physical and social frailty in participants in a frailty prevention program during the COVID-19 pandemic. Methods: A cross-sectional survey was conducted in Japan from January 2021. Further, 863 participants of a frailty prevention program completed the survey. The frequency of program attendance in 2020, physical frailty (5-item frailty screening index), social frailty（diagnostic criteria of social frailty in NCGG-SGS), and depression (GDS-5) were assessed. A related factor of physical frailty was analyzed statistically by Welch's t test and the Chi-squared test. Results: The study participants’ mean age, proportion of women, and mean enrollment period in program were 86.8±4.9, 96.3%, 64.3±48.6 months, respectively. The program attendance ratio was 83.4% from January to March, 54.5% from April to June, 79.8% from July to September, and 80.0% from October to December. The prevalence of physical frailty was 20.3% non-frailty, 63.7% pre-frailty, and 15.6% frailty. The prevalence of social frailty was 10.0% non-frailty, 28.6% social pre-frailty, 61.8% social frailty, and the prevalence of depression was 36.8%. Participants with physical frailty were significantly older and showed higher prevalence of social frailty and depression, displaying significantly lower attendance program than non-frailty and pre-frailty older adults (p<0.05). Conclusions: The study results suggest that more than half of the participants of a frailty prevention program have social frailty and a high risk of physical frailty due to COVID-19.

